# Liquid Biopsy as a Tool Exploring in Real-Time Both Genomic Perturbation and Resistance to EGFR Antagonists in Colorectal Cancer

**DOI:** 10.3389/fonc.2020.581130

**Published:** 2020-09-25

**Authors:** Valeria Internò, Marco Tucci, Gaetano Pezzicoli, Franco Silvestris, Camillo Porta, Francesco Mannavola

**Affiliations:** ^1^Department of Biomedical Sciences and Clinical Oncology, University of Bari Aldo Moro, Bari, Italy; ^2^National Cancer Research Centre, Istituto Tumori Bari “Giovanni Paolo II”, Bari, Italy

**Keywords:** liquid biopsy, colorectal cancer, EGRF, panitumumab, cetuximab, resistance

## Abstract

The treatment of metastatic colorectal cancer (mCRC) has improved since the introduction of the epithelial growth factor receptor (EGFR) inhibitors as cetuximab and panitumumab. However, only patients with peculiar genomic profiles benefit from these targeting therapies. In fact, the molecular integrity of *RAS* genes is a predominant factor conditioning both primary and acquired resistance in non-responders although additional molecular derangements induced by selective anti-EGFR pressure may concur to the failure of those disease treatment, liquid biopsy (LB) appears as a surrogate of tissue biopsy, provides the genomic information to reveal tumor resistance to anti-EGFR agents, the detection of minimal residual disease before adjuvant therapies, and the discovery of tumor molecular status suitable for rechallenging treatments with EGFR antagonists. LB investigates circulating tumor cells (CTCs), cell-free tumor DNA (ctDNA), and tumor-derived exosomes. In mCRC, ctDNA analysis has been demonstrated as a useful method in the mutational tracking of defined genes as well as on tumor burden and detection of molecular alterations driving the resistance to anti-EGFR targeting treatments. However, despite their efficiency in molecular diagnosis and prognostic evaluation of mCRC, the affordability of these procedures is prevalently restricted to research centers, and the lack of consensus validation prevents their translation to clinical practice. Here, we revisit the major mechanisms responsible for resistance to EGFR blockade and review the different methods of LB potentially useful for treatment options in mCRC.

## Introduction

During the past 20 years, the adoption of monoclonal antibodies (mAbs) targeting the epithelial growth factor receptor (EGFR) has led to a dramatic improvement in the survival of metastatic colorectal cancer (mCRC) patients bearing RAS in its wild type isoform (RASwt). However, extensive data from clinical trials conducted in the first-line setting showed that approximately 75% of early responders to EGFR blockade undergo tumor progression within 12 months, while 20% of patients are primary resistant and only less than 5% are actually long-time responders (1–5).

Therefore, to optimize therapeutic response for the management of mCRC, it would be key to identify molecular mechanisms able to induce both primary and acquired resistance to anti-EGFR mAbs, detect pre-existing gene alterations, and monitor the onset of *de novo* abnormalities restraining cancer sensitivity to anti-EGFR mAbs.

Recent studies highlighted the mutations of BRAF (B-raf proto-oncogene serine/threonine kinase) and PIK3CA, as well as the amplification of HER2/MET, among major events driving resistance to anti-EGFR treatments (6, 7). However, these studies were mainly conducted on tumor biopsies obviously requiring invasive procedures, often limiting the genomic analysis of the tumor to a single snapshot of a few cells (8). In addition, the measurement of molecular patterns in tissue biopsies does not represent the real-time molecular state of the tumor, and the dynamic changes adopted by tumor cells to escape the selective pressure of anti-neoplastic drugs.

In this contest, liquid biopsy (LB) has emerged as an alternative test able to provide, during the course of treatment, a tumor’s actual molecular profile, namely a real-time gene assessment. LB is based on the detection and isolation of tumor-derived components from body fluids, including nucleic acids, circulating tumor cells (CTCs), and extracellular vesicles (EVs); overall, it is a minimally invasive test easily providing the molecular snapshot of a given tumor (9). Furthermore, this procedure has many potential applications in CRC including early diagnosis, detection of minimal residual disease, concurrent molecular assessment, prognostic stratification, and monitoring the response during treatments (10–13). It may also provide real-time monitoring of the clonal evolution of a tumor during its treatment, early detect the development of resistant clones, and unmask disease progression much earlier with respect to conventional radiological procedures. Recent technological improvements have increased its sensitivity, thus allowing the detection of minimal numbers of cancer cells harboring molecular defects associated with resistance to EGFR blockade. To this regard, LB using as substrate the cell-free tumor DNA (ctDNA) has provided considerable application in tracking the RAS mutational (RASmut) status, in order to refine the use of anti-EGFR mAbs in CRC, while a limited experience exists to date regarding either CTCs or EVs. Thus, based on both scientific impact and suitability of this procedure, a number of clinical trials are presently evaluating possible applications of ctDNA obtained by means of LB in the management of mCRC patients (14–16), although some unmet needs are still evident, due to the lack of standardized methods and optimization of pre-clinical variability.

Here, we discuss the role of LB in investigating the mechanisms driving resistance to anti-EGFR therapies and review the most recent clinical trials exploring its possible impact on mCRC management.

## Molecular Mechanisms of Resistance to Anti-EGFR mABs

**FIGURE 1:**
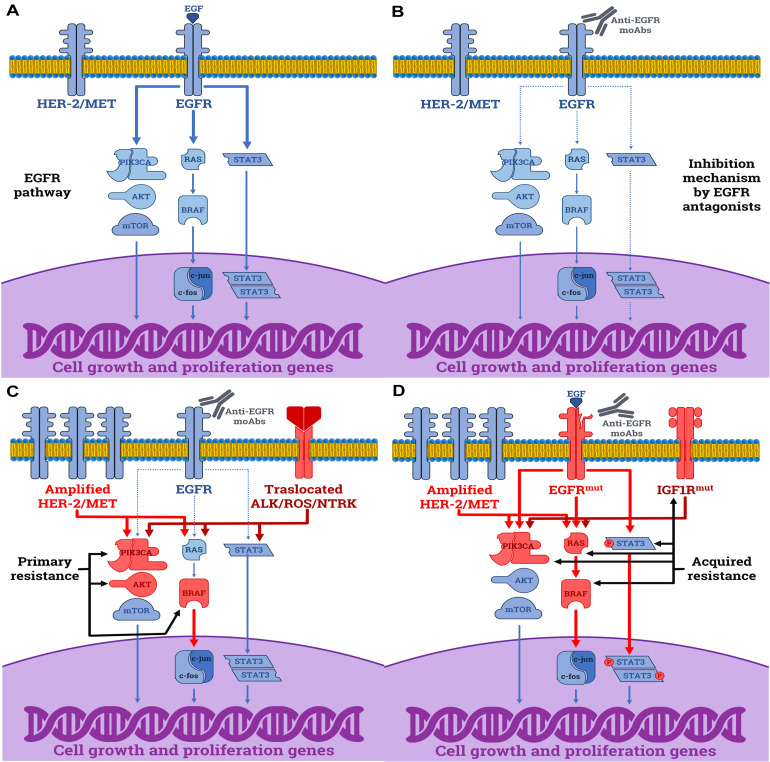
Molecular mechanisms driving the resistance to anti-EGFR mAbs in CRC cells. **(A)** The normal function of EGFR by EGF leading to the activation of downstream proliferative signals (continuous arrows). **(B)** Anti-proliferative effects induced by cetuximab and panitumumab in sensitive RASwt CRC cells by disabling the downstream cascade of the EGFR (dashed arrows). **(C)** Primary resistance mechanisms to anti-EGFR mAbs in RASwt cells include: (i) activating mutations of downstream elements as BRAF, PIK3CA, and AKT; (ii) amplification of HER2 or MET receptors; (iii) rearrangements of ALK, ROS, RET or NTRK receptors. **(D)** Acquired resistance mechanisms to anti-EGFR mAbs are: (i) mutations affecting the epitope of EGFR recognized by mAbs; (ii) activating mutations in downstream elements, including BRAF, PIK3CA, or RAS genes; (iii) STAT3 phosphorylation; (iv) activation of parallel growth factor receptors (HER2/MET amplifications or IGF1R activating mutations). The blue elements are normal functioning proteins or receptors, while those in red derive from gain-of-function mutations.

Understanding the molecular mechanisms that underly both primary and acquired resistance to anti-EGFR mAbs is mandatory to optimize treatment decisions in mCRC, and the pre-existing RASmut status has been repeatedly described as the predominant event responsible of therapeutic failure to anti-EGFR mAbs in RASmut patients (17, 18). However, RASmut is not the unique mechanism able to overcome the sensitivity to EGFR blockade, since several other molecular alterations have been described. Several derangements of the major pathways involved in generating both primary and acquired resistances are next described and summarized in Figure 1.

### Primary Resistance

Two mechanisms have been proposed to drive primary resistance. The first one depends on native gene mutations, not related to RAS, that independently from the EGFR downstream machinery activate the MAPK pathway signals. The other is based on the activation of alternative pathways (19).

BRAF is a serine-threonine kinase involved in the downstream signaling of EGFR. Mutations affecting BRAF and RAS are mutually exclusive and occur in about 5–20% of mCRC (20). Noteworthy, BRAFmut mCRCs include a distinct subset of aggressive tumors that are frequently located within the right colon and are associated with a defective mismatch repair (dMMR) system or elevated mutational tumor burden (21). Similarly to melanoma, the majority of BRAFmut polymorphic variants hit the V600 codon, thus resulting in the constitutive activation of the MAPK cascade with consequent inefficacy of EGFR blockade, and poor responsiveness to systemic therapies (20–22). The failure of anti-EGFR treatments in these patients is also confirmed by the major effectiveness of intensified treatments such as “FOLFOXIRI regimen” to restrain the tumor progression, although the predictive role of BRAFmut has not been defined in the response to cetuximab or panitumumab (23). Since the possible benefits deriving from the combination of anti-EGFR agents to chemotherapy are still debated in this setting, the mutational analysis of BRAF is not currently used in clinical practice, although promising results have been reported by combining BRAF/MEK inhibitors with anti-EGFR agents such as cetuximab (24).

Mutations affecting the Phosphatidylinositol-4,5-bisphosphate 3-Kinase Catalytic Subunit Alpha (PIK3CA), namely a signal transducer downstream of activated cell surface growth factor receptors, have also been implicated in the primary resistance to anti-EGFR agents, and are described in 3–5% of patients. In fact, germline mutations affecting exon 20 are able to induce a poor response to cetuximab or panitumumab, as a result of the hyperactive proliferation signaling which is propagated independently from EGFR activation. By contrast, activating mutations of PIK3CA on exon 9 strictly depend on the EGFR cascade, and require the interaction with RAS proteins, thus maintaining the responsiveness to EGFR blockade (6).

Further molecular events driving primary resistance to anti-EGFR mAbs involve the native activation of alternative patterns, such as HER2/PTEN and AKT1 mutations, HER2/MET amplifications, or NTRK/ROS/ALK/RET rearrangements (25, 26). Although these molecular alterations are rare in CRC (1–5%), their frequency increases within the RAS-BRAFwt population and may be assessed in this subgroup of patients to predict the favorable response to anti-EGFR blockade. In fact, these rare genetic alterations have been investigated to evaluate the therapeutic response to both cetuximab- or panitumumab-containing protocols in a prospective case-control study, and at least one of these abnormalities has been detected within the non-responders with a significant prevalence (42.6%) (7).

### Acquired Resistance

A percentage as high as up to 60% of patients develop disease progression during treatments as a result of acquired resistance to anti-EGFR antagonists. Therefore, relative molecular switches and/or derangements may become new potentially druggable targets to prevent cancer cell escape from the EGFR-signaling blockade. Indeed, novel mutations within the binding domain of EGFR have been described to impair the interaction between this receptor and cetuximab, thus greatly limiting its efficacy. Other mechanisms interacting with the parallel downstream signaling are reported to induce hyperactivity of the MAPK cascade and other RAS-independent pathways, including STAT3 phosphorylation, and IGF1 activation, or to generate *de novo* mutations of RAS, BRAF, and PIK3CA as well as HER2/MET amplifications (27). However, these mechanisms may be concomitantly activated and accumulate as a direct consequence of selective anti-EGFR pressure which primes the expansion of sub-clones constitutively resistant to EGFR antagonists (28). Based on this limited information on CRC genomic heterogeneity both in germline and somatic development, before planning treatments with anti-EGFR blockers, it would be critical to investigate the dynamic molecular landscape of CRC in order to suggest the most suitable and hopefully efficient therapy to each patient in a precision medicine environment.

## Liquid Biopsy in CRC Clinical Management

**FIGURE 2:**
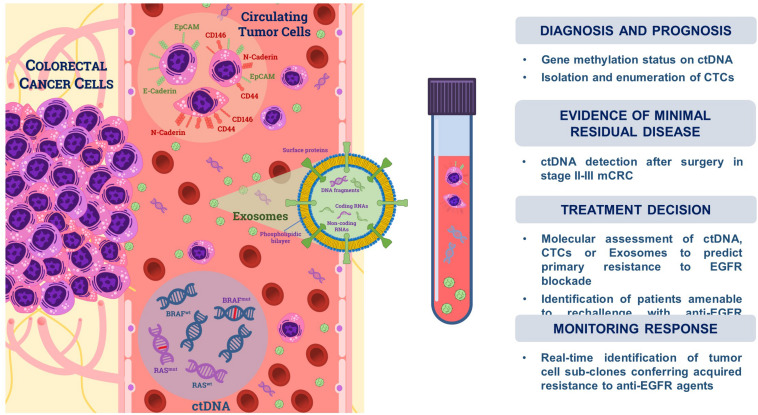
Liquid biopsy in colorectal cancer. Tumor cells undergoing the epithelial-to-mesenchymal transition (EMT) are shed from primary tumors and can be isolated from the bloodstream as circulating tumor cells (CTCs). They variably express either epithelial (e.g., EpCAM, E-Cadherin) or mesenchymal markers (e.g., CD44, CD146, and N-Cadherin). Tumor cells also release DNA fragments (<200 bp) through apoptosis, necrosis, or active secretion, namely cell-free tumor DNA (ctDNA), whose fraction in the blood circulation ranges from 0.01 to 90% with respect to non-tumoral cell-free DNA (cfDNA). Exosomes (Exo) are small extracellular vesicles (<200 nm) actively secreted by cancer cells which are composed by a phospholipidic bilayer encapsulating proteins, coding- and non-coding RNAs as well as DNA fragments. Isolation from blood circulation and downstream analysis of these tumor-derived components may be used for both diagnostic as well as prognostic or predictive purposes in CRC, as summarized on the right side of the panel.

Translational studies exploring the possible use of LB in CRC have rapidly increased in the last decade and both quantitative and qualitative contributions were derived from analysis of CTCs, ctDNA, and exosomes (Exo) (29), which are directly released by tumors in the bloodstream and can be thus easily detected (Figure 2).

### Circulating Tumor Cells

Circulating tumor cells are cancer cells detached from the primary tumor and disseminated into the bloodstream to reach distant sites in which they may generate metastasis. They have been primarily proposed for quantitative analysis in breast cancer to monitor prognosis and response to treatment, and their peripheral counts are currently considered a suitable biomarker of cancer activity in a number of tumors. To date, only the CellSearch© device has been approved for CTC detection by the U.S. Food and Drug Administration (FDA). This system recognizes CTCs by recovering the epithelial surface marker EpCAM through dedicated immunomagnetic adsorption and live cytometry imaging (30). The peripheral CTC count by CellSearch© is currently approved to measure the therapeutic response in breast, colon, and prostate cancers. In mCRC, the detection of ≥3 EpCAM+ CTCs in 7.5 ml of venous blood after treatments is considered an independent factor that correlates with unfavorable prognosis and short survival (31, 32). Despite this evidence, the CTC count is rarely used for the clinical management of mCRC for several reasons including the need for expensive equipment, the time needed to perform the test, not to take into account the fact that it does not provide clear-cut therapeutic indications, thus not providing a real benefit for patients (33).

**FIGURE 3:**
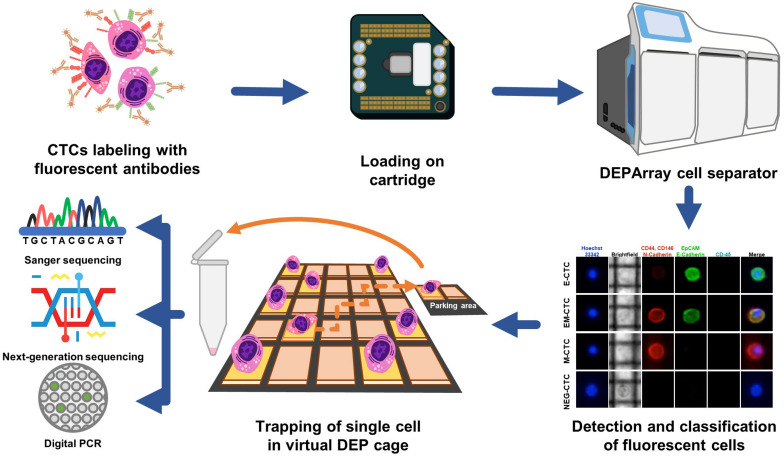
Isolation of CTCs using the DEPArray system. Circulating tumor cells isolated from peripheral blood are labeled with specific fluorochrome-conjugated antibodies against typical EMT markers. Cells are loaded in a dedicated cartridge and visualized by fluorescence microscopy. As represented in the fluorescence panel, CD45neg CTCs variably express epithelial (EpCAM, E-Cadherin) or mesenchymal (N-Cadherin, CD146, and CD44) markers although a small amount of these cells may share markers of both lineages. This method also allows the size evaluation of CTCs in brightfield. Thus, CTCs are moved into a parking area using dielectric forces and recovered as single or clamped cells. Further downstream molecular analyses are next assessed on these samples by either Sanger sequencing, NGS, or dd-PCR.

Recently, an innovative system (DEPArray) was developed to allow both detection and sorting of single CTCs by either surface or cytoplasmic markers and by separation of cells in relation to their dimensional and dielectrophoretic movimentation properties as depicted in Figure 3 (34). This methodology, however, appears more fruitful for qualitative analysis of the genomic assessment of mCRC since, after the identification of the cancer phenotype, it may isolate also single cells. Thus, differently from CellSearch©, the DEPArray can be used to detect CTCs expressing not only EPCAM, but also mesenchymal markers typically occurring in epithelial-to-mesenchymal transition (EMT), such as CD44, CD146, vimentin, or N-Cadherin, which characterize the metastatic phenotype (35). Hence, different subpopulations of CTCs are potentially detectable by the DEPArray system in relation to tissue-specific or cancer-associated functional markers (36), which otherwise would not be appreciable using CellSearch©.

Despite limited experience in mCRC setting (37), to date DEPArray has been proven able to enrich the pool of CTCs which include differently functional subsets as recently proposed in breast cancer by Bulfoni et al. (38). Alternative methods for CTC detection are based on the recognition of mRNA markers typically expressed by either epithelial- or mesenchymal-like neoplastic cells or on the conjugation of negative selection with physical measurements (39, 40). In this contest, the AdnaTest combines the EPCAM-based enrichment of CTCs with the recognition of EpCAM, EGFR, and CEA transcripts using an RT-PCR approach (41).

Mutational analyses of CTCs are not currently supported in clinical practice. However, their isolation and downstream molecular profile assessment may provide useful information for planning adequate strategies, or evaluating the response to therapies. Recent advancements in Next Generation Sequencing (NGS) technology, allowing deep genotyping at the single-cell level, have emphasized the high heterogeneity of CTCs (42), thus adding complexity in our attempt to translate these techniques into everyday clinical practice.

### Cell-Free Tumor DNA

Cell-free DNA (cfDNA) fragments (<200 bp) are released into the bloodstream as a result of cell apoptosis, necrosis, or shedding during pathological and physiological conditions such as exercise, trauma, renal failure, stroke and cancer (43). In addition to the passive release of their own DNA fragments, cancer cells actively spread ctDNA for several functions related to tumor progression as cell-to-cell crosstalk, distant molecular commitment or preparation of the metastatic niche (44). Although ctDNA is only a fraction of the cfDNA (0.01–90%), it is considered a surrogate for tumor genome, suitable for many molecular tests, such as mutational tracking, measurement of tumor burden, as well as DNA methylation and assessment of microsatellite instability (MSI) (45).

Highly sensitive and reproducible detection methods can discriminate ctDNA from cfDNA and are thus categorized as “targeted” or “non-targeted” approaches. The polymerase chain reaction (PCR)-based method used in early studies has been replaced by highly sensitive digital PCR techniques, such as droplet digital (dd)-PCR or BEAMing (beads, emulsion, amplification, and magnetics) technology (46–48). They are suitable for the detection of tumor-specific (targeted) mutations with high sensitivity (0.001%), although the number of genes that can be simultaneously assessed is restricted. On the contrary, novel NGS technologies including TAm-Seq (tagged amplicon deep sequencing), Safe-Seq (safe sequencing system), and CAPP-Seq (cancer personalized profiling by deep sequencing), are suitable for concomitantly investigating larger numbers of genes (49, 50). Nevertheless, these methods are based on the mutational tracking approach and prevalently investigate known mutations in ctDNA, whereas unknown molecular defects located in DNA regions that are not checked, may thus remain undetected in the same ctDNA sample.

“Non-targeted” approaches such as the Whole Genome Sequencing (WGS) and Whole Exome Sequencing (WES) allow to discover *de novo* mutations and detect structural rearrangements, gene fusions, copy number variations, and other genomic derangements. Although no preliminary information on tumor genome is required, a large amount of cfDNA is needed for a reliable reconstruction of the tumor-specific genome-wide changes, thus limiting the applicability of this tool in clinical practice (51, 52). More recently, several researchers have focused on ctDNA methylation as a potential marker for CRC diagnosis, since methylation changes occur early along with the natural history of a given tumor, and are restricted to defined genomic loci (53–55). To this, Barault et al. presented a dd-PCR five-gene methylation panel (EYA4, GRIA4, ITGA4, MAP3KI4-ASI, and MSC) on ctDNA to overcome the absence of patient-specific mutations for LB (56). Similar findings were obtained by studying the methylation status of two other genes on cfDNA, namely the WNT inhibitory factor 1 (WIF1) and Neuropeptide Y (NPY) (57, 58). These methods are very versatile for their potential prognostic use, as well as for monitoring tumor burden, or the response to treatment. Notably, in a recent pre-planned analysis of the large IDEA-FRANCE phase-III trial, post-surgical detection of methylated ctDNA in stage III CRC was found to be an independent negative prognostic factor of recurrence, thus proposing its clinical application in the adjuvant setting (59).

Finally, quantitative ctDNA analysis is usually considered for monitoring tumor burden, or as a bio-marker of anti-cancer treatments. An increased amount of ctDNA directly reflects the tumor progression as an effect of active proliferation of drug-resistant cancer cells, whereas the reduction of ctDNA fraction occurs after surgery, or in response to effective treatments. With respect to CRC, as well as to other tumors, the detection of ctDNA in stage II, may reflect the presence of minimal residual disease, thus identifying patients at high risk of tumor recurrence (60, 61).

### Exosomes

Another emerging tool in this evolving scenario includes the possibility to isolate and investigate EVs directly shed by cancer cells. These particles of variable diameter, are formed by a phospholipid bilayer and can be found in all biologic fluids, such as blood, urine, saliva, and ascites (62). Those bearing a nano-sized diameter (30–100 nm) are currently referred to as small-EVs or Exo (63, 64). They are involved in intercellular communication by delivering cargos of active molecules including proteins, messenger RNAs, non-coding RNAs, and DNA fragments from one to another cell (65). Many studies in CRC revealed that Exo are involved in tumor progression and metastasis, as well as in tumor resistance against either cytotoxic or targeting agents (66, 67).

Differently from cell-free RNA (cfRNA) or cfDNA, the nucleic acids packaged within the phospholipid bilayer of Exo are protected from the degradation by serum ribonucleases and DNases and are easily accessible for downstream analyses. Peculiar interest in this field has been devoted to the study of exosomal micro-RNAs (miRNAs) whose defective levels in CRC are currently under investigation for both prognostic and predictive purposes (68). Moreover, other potentially useful applications include the possibility to isolate and analyze Exo released by immune cells. Their phenotypic profile indeed may reflect the immune system’s activity against the tumor, as already demonstrated during immunotherapy of melanoma (69).

Despite the potentiality of Exo as a suitable substrate for LB, a strong limitation for their use derives from the lack of a standardized procedure for the purification of nanovesicles and further analyses. Some commercial kits are already available for rapid and easy purification of Exo, but a suitable yield requires expensive and time-consuming ultracentrifugation of large volumes of biological fluids. This has hence limited the applicability of Exo as a high-throughput diagnostic tool.

## Liquid Biopsy and anti-EGFR Therapy

The possibility to predict and evaluate in real-time the actual response to anti-EGFR agents is the most relevant application of LB in mCRC, while the identification of patients potentially amenable to rechallenge treatments also represents a goal pursuable by LB. Table 1 summarizes recent studies with ctDNA, CTCs, and Exo that addressed these aims.

**TABLE 1:** Major studies exploring LB in mCRC for molecular testing.

**Studies**	**Genes tested**	**Detection method**	**Findings**
*ctDNA*			
(70)	*KRAS*	Droplet digital-PCR	Sensitivity 87%; specificity ∼100%
(72)	*BRAF*	ARMS-qPCR	100% concordance with tissue biopsy
(71)	*KRAS*	NGS	80% concordance with tissue biopsy; *KRAS*^*mut*^
(CAPRI-GOIM trial – *Phase II*)			correlate with primary resistance to EGFR blockade
(82)	*KRAS*	PCR assay	The occurrence of *RAS*^*mut*^ reflects the clonal evolution
			Of CRC during EGFR blockade and the onset of
			molecular mechanisms responsible of acquired
			resistance to cetuximab or panitumumab
(73)	226 gene panel	BEAMing	Several alterations undetected with tissue biopsy were revealed on ctDNA of patients with primary resistance to anti-EGFR agents. *RAS*^*mut*^ emerge during EGFR blockade and decline upon drug withdrawal
(84)	*RAS, BRAF*	dd-PCR ultra-deep NGS	Persistence of *RAS*^*mut*^ on ctDNA before third-line
(CRICKET trial – *Phase II*)			rechallenge with cetuximab is associated with treatment failure
NCT03227926	*RAS*	NGS	This ongoing trial will explore the efficacy of a third-
(CHRONOS trial – *Phase II*)			line rechallenge with panitumumab in a *RAS*^*wt*^ mCRC cohort of patients selected on the basis of *RAS* extended ctDNA clonal evolution
*CTCs*			
(75)	*KRAS, BRAF, PIK3CA*	*Vortex Gen1 and Sanger Seq*	High (∼80–90%) concordance with tissue biopsy
(37)	*KRAS*	*DEPArray system and WGA*	
(77)	EGFR (mRNA)	CellSearch© and MagNest	EGFR expression did not correlate with survival in
(78)	EGFR, CEA and EPCAM (mRNA)	AdnaTest (Colon Cancer)	*RAS*^*wt*^ mCRC treated with cetuximab
(13)	*KRAS, NRAS, BRAF*	CellSearch© and ddPCR	High (97%) concordance of basal mutational status between tumor biopsies, CTCs and ctDNA. *De novo* mutations conferring acquired resistance to anti-EGFR drugs were earlier detected by CTCs than ctDNA
(83)	50 genes included in the	LFIMA and NGS	In a small cohort of RAS^wt^ mCRC patients undergoing
	AmpliSeq Cancer Hotspot		anti-EGFR therapy, *de novo* mutations of *SMARCB1*,
	Panel (Thermo Fisher)		*EGFR, ATM, and PIK3CA* genes were detectable on CTCs (but not on ctDNA)
*Exosomes*			
(79)	*KRAS, BRAF*	Serum ultracentrifugation	Sensitivity 73–75%
		and PCR-based sequencing	Almost 100% concordance with tumor tissue
(80)	UCA1-lncRNA levels	Serum ultrafiltration and	Increased levels of exosomal UCA1 in *RAS*^*wt*^ mCRC pts
		qRT-PCR	were correlated with primary resistance to cetuximab

### Predicting Primary Resistance

Liquid biopsy has been recently investigated as an alternative tool for RAS mutational analysis on CRC tissue specimens. In a recent study in mCRC, Bettegowda et al. proved that the sensitivity of dd-PCR to detect mutations of KRAS in ctDNA was as high as up to 87.2%, with specificity equal to 100% (70). Although the number of patients analyzed in this study was quite limited (*n* = 12), these results prompted further analysis to validate the ctDNA as a surrogate of tissue biopsy. In fact, in the CAPRI-GOIM trial, the RAS molecular testing was performed by NGS and demonstrated a concordance near 80% between tissue and plasma samples. The early detection of KRASmut in ctDNA in chemotherapy-free patients was correlated with primary resistance to anti-EGFR mAbs, while both objective response rate (ORR) and overall survival (OS) were improved in patients harboring a wild-type status of RAS in ctDNA (71).

Similarly, Spindler et al. found a linear agreement of the BRAF status between ctDNA and tissue samples (72). The same authors revealed a strong predictive and prognostic value of both the RAS and BRAF molecular status on ctDNA as compared to tumor biopsy. However, the high molecular intratumoral heterogeneity observed as well as the small sample size and the non-randomized design of the study strongly limit definitive conclusions.

By using a massive NGS of 226 genes, moreover, Siravegna et al. retrospectively analyzed the basal ctDNA mutational landscape of patients with RASwt mCRC that were refractory to anti-EGFR agents, to discover defects putatively correlated to primary resistance (73). Noteworthy, in 50% of patients, they found new molecular aberrations that were associated with intrinsic resistance to panitumumab or cetuximab such as alterations of ERBB2, FLT3, EGFR, and MAP2K1. These findings suggest that ctDNA is a broadly applicable, sensitive, and specific biomarker that may capture intra-patient disease heterogeneity.

Several commercial kits for ctDNA analysis are available to date, such as the OncoBEAM RAS CRC Kit (Sysmex Inostics) and the Idylla ctKRAS/ctNRAS-BRAF Mutation Test (Biocartis) which received the European approval for detection of RAS/BRAF mutations in CRC (74). However, there is not enough clinical evidence to establish the percentage threshold of mutated RAS alleles on ctDNA that confers resistance to anti-EGFR therapy. Therefore, analysis of RAS status for therapeutic decisions should still be performed on tumor tissue, while LB would be used in case of insufficient or inaccessible tissue. The analysis of CTCs has been also considered as an alternative tool for molecular profiling of mCRC patients. Detection of KRAS and BRAF mutations in CTCs is challenging, but feasible, with almost 10–20% rate of discordance between primary and metastatic tumors (37, 75). However, data on the predictive value of the molecular analysis of CTCs are still lacking, while pioneering works on the use of CTC count or the expression of EGFR by CTCs did not meet the expected results (76–78).

Finally, Hao et al. assayed the KRAS/BRAF mutational status of serum Exo from metastatic CRC patients, and found a high concordance with primary tumors (79). Further studies are nevertheless needed before considering this approach as a reliable alternative to molecular testing on tissue biopsy since these results may have been biased by serum sample preparation or Exo collection, as well as by methods applied for DNA extraction or sequencing. An additional study recently proposed the measurement of serum exosomal UCA1-lncRNA levels to identify patients with RASwt mCRC primarily resistant to anti-EGFR mAbs (80). This is an excellent example of the wide potentiality of Exo to investigate epigenetic mechanisms that regulate both intrinsic and acquired resistance to targeted therapies, even if there are no standardized methods for their isolation and downstream analysis.

### Monitoring Treatment Response

The methods approved to evaluate the response to therapies in oncology are based on morphological (CT or MRI), or metabolic (18FDG-PET/CT) modifications of neoplastic target lesions, mainly using the Response Evaluation Criteria In Solid Tumors (RECIST) or PET Response Criteria In Solid Tumors (PERCIST) criteria (81). These traditional methods, however, do not allow to appreciate in real-time the actual onset of resistant clones with a consequent possible delay in the identification of drug resistance. LB may overcome these limitations and forestall the radiological progression. In particular, the onset of *de novo* KRAS or NRAS mutations in ctDNA were able to predict the resistance to anti-EGFR agents in different studies enrolling RASwt mCRC patients (10–12). A possible explanation is that colorectal tumors harboring RASwt gene sequences include a minimal compartment of mutated clones before therapy. The amount of these cells hence increases for the selective pressure exerted by anticancer drugs, until the amount of RASmut clones prevails on the wild-type counterpart, and patients become thus unresponsive to EGFR blockade (82).

The possible applications of cfDNA and CTCs have also been investigated. Sun et al. explored the mutational status of KRAS, NRAS, and BRAF genes in CTCs and ctDNA from blood samples of patients harboring RAS/BRAFwt mCRC from the beginning of treatment (13). They found about 97% concordance of the basal mutational status of tumor samples, CTCs and ctDNA while 40% of patients acquired *de novo* mutations within 9 months from the initial EGFR blockade. However, half of these acquired mutations were intermittent, independent from therapeutic changes, thus questioning their clinical validity in the course of treatment. Patients developing new mutations in CTCs or ctDNA at the disease progression were characterized by a worse prognosis with a 12-months median OS, as compared to 20-months of those harboring the wild-type status. Furthermore, these mutations were earlier detectable in CTCs with respect to ctDNA, based on the ability of tumor sub-clones to rapidly spread into blood circulation while ctDNA shedding from necrotic and apoptotic cells occurs later. By using a label-free inertial microfluidics approach (LFIMA) for CTC isolation, Onidani et al. optimized an NGS-based method for analyzing a wide spectrum of gene mutations. Although a small number of mCRC patients were receiving anti-EGFR therapy, they monitored the emergence of new alterations along with treatment. Notably, newly acquired mutations affecting several genes, such as SMARCB1, EGFR, ATM, and PIK3CA, were demonstrated on CTCs as the disease progressed. The majority of these alterations were not detectable in ctDNA at the same time point and, therefore, they concluded that CTCs should be an optimal tool for the early detection of *de novo* mutations, although simultaneous assay on ctDNA should be provided (83).

**FIGURE 4:**
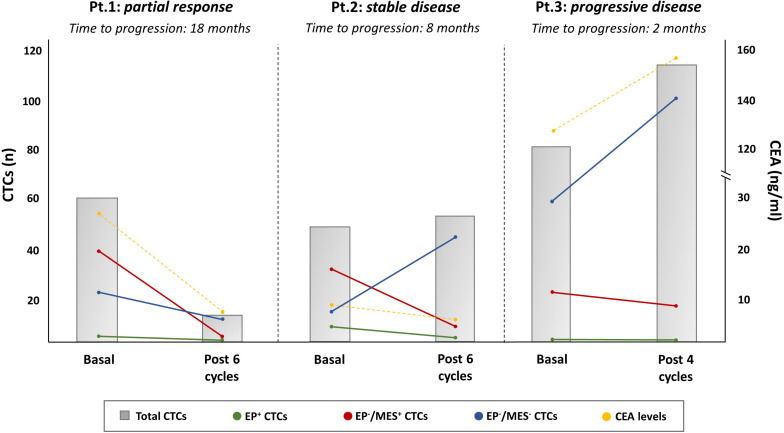
Longitudinal monitoring of CTCs by EMT marker expression in mCRC patients in relation to the therapeutic response to EGFR antagonists. The picture is representative of three RASwt/BRAFwt mCRC patients whose CTC counts and phenotypical distributions, both at baseline and at the first evaluation, varied after treatment with FOLFOX plus PANITUMUMAB (Pt. 1 partial response, Pt.2 stable disease, and Pt.3 progressive disease). DEPArray System was adopted for CTCs capturing by measurement of typical epithelial (EpCAM, e-Cadherin) and mesenchymal (CD44, CD146, and N-Cadherin) markers. As shown, Pt.1 experienced a partial response and, consequently, the reduction of CTC amounts and different phenotypical populations (MES+ and EP-/MES – CTCs, namely double negative CTCs). In Pt.2, whose mCRC was stable after six cycles of therapy, the total count of CTCs remained unchanged with a different distribution of phenotype populations. In this case, the number of double negative CTCs increased with a parallel reduction of MES+-CTCs. In Pt.3, a patient with primary resistance disease, the CTC count, including both double negative and MES+-CTCs increased, in a similar fashion as for CEA. All three patients showed a minimal level of EP+-CTCs supporting the relevance of CTC purification to detect those with phenotype different from the epithelial one. The gray bars are the total CTC counts, while colored dots represent EP+ (green), EP–/MES+ (red), and EP–/MES– (blue) CTCs, respectively; CEA levels were also measured (yellow dots).

Despite CTCs may have not a direct application for personalizing treatments, they offer a proof-of-concept for testing the onset of newly molecular alterations associated with acquired resistance to anti-EGFR agents. This could be helpful for the early detection of patients who probably benefit from a rapid switch to alternative drug schedules. However, an adequate number of CTCs is required for this purpose and the CellSearch© does not seem the optimal tool for CTC downstream analysis due to few EpCAM + cells detectable in the blood circulation. By contrast, the DEPArray system appears to be more suitable for this purpose, since it allows the capture of a wide number of CTCs with variable expression of EMT marker (35, 36). Moreover, it is conceivable that the amount of CTCs with different EMT phenotypes is representative of variable sensitivity to treatments, while their modifications along with the use of anti-EGFR agents may reflect response, as we have recently experienced at our institution using the DEPArray (Figure 4).

### Selection of Patients Amenable for Anti-EGFR Rechallenge

The possible utility of re-using EGFR inhibitors after discontinuation due to initial tumor progression is a hot topic in mCRC. In the CAPRI-GOIM trial, 182 tumor samples from KRASwt (exon 2) mCRC were retrospectively examined by NGS to identify a subset of patients who benefited from rechallenge with cetuximab (71). This study showed that only patients harboring at baseline KRAS (exons 2, 3, and 4), NRAS, BRAF, and PIK3CA wild-type tumors had a minimum advantage in terms of PFS when rechallenged. However, although encouraging, this study failed to demonstrate a predictive value of basal tissue biopsy since both molecular heterogeneity and clonal evolution of CRC induced by the selective pressure from anticancer treatment obviously generated several genomic modifications that were not present at baseline. A step forward in this field was moved by Siravegna et al., who described, with the limits of a retrospective analysis, a “pulsatile behavior” of KRASmut along with the EGFR blockade that increased during treatment, while rapidly decreased after drug discontinuation (73). This trend over time of KRASmut in ctDNA may suggest re-challenging anti-EGFR agents in the next lines of therapy. Thus, the early identification of acquired resistance leads to optimization of the duration of EGFR blockade, justifying treatment interruption if *de novo* RASmut are detected in ctDNA and its recovery after ctDNA normalization. In this context, the CRICKET trial prospectively assessed the efficacy of anti-EGFR agents as a third-line treatment for patients with RAS/BRAF wild-type mCRC who were initially sensitive to first-line cetuximab-based therapy. In this study, 28 patients underwent rechallenge with cetuximab, and response in more than one half of them was reported. To retrospectively characterize patients who benefited from rechallenge, blood samples were collected before restarting cetuximab and ctDNA verified by dd-PCR ultra-deep NGS for RAS and BRAF mutations. The RASmut was found in ctDNA collected at the time of rechallenge start in 12 out of 25 patients, whereas RASmut was not detected in those achieving a partial response. Moreover, patients bearing RASwt ctDNA had a significantly longer median PFS than RASmut (4.0 vs. 1.9 months) (84).

Finally, another possible contribution in this field is expected from the ongoing Italian CHRONOS phase II trial (NCT03227926) (14). This is a prospective liquid biopsy-driven study exploring the use of RAS mutations in ctDNA of mCRC patients to predict the efficacy of third-line rechallenge with panitumumab. Unlike previous studies, patients are considered eligible for rechallenge only in the presence of a consistent reduction of RASmut ctDNA fractions from the withdrawal of first-line chemotherapy with anti-EGFR to the time of rechallenge. Moreover, tumor ctDNA will be assayed by NGS either before and after panitumumab rechallenge to identify potential associations between different molecular alterations rather than RAS and response to rechallenge.

## Conclusion

In the era of precision medicine, the modern oncology is aimed at identifying personal treatments for each patient that are suitable for the molecular signature of relative tumors. Precision medicine would indeed be necessary to select patients for molecularly targeted therapies, for longitudinal monitoring of treatment response and for exploring the clonal evolution of cancer cells along with the treatment. In this context, LB has gained increasing interest as a simple method aimed at evaluating either functional or inactive tumor-released components from which obtaining a real-time molecular snapshot of cancer. However, although major advances have been reached by investigating CTCs particularly in breast and prostate cancers, in mCRC the LB on ctDNA has recently provided results suitable for its future translation to the clinical practice.

Particularly in mCRC, the use of LB on ctDNA has been recently adopted to discover minimal residual disease before adjuvant treatments, as well as for detecting molecular genomic derangements predictive of primary or secondary resistance to anti-EGFR agents. The pulsatile behavior of RASmut in ctDNA also paves the way for dynamic monitoring of treatment response, or identification of candidates eligible to EGFR blockade rechallenge. The modern technology of DNA sequencing supports these purposes since they are endowed with high sensitivity and moderate costs.

Nowadays, the unsolved limits before a definitive validation of LB in the clinical practice are mostly due to the lack of standardized methods as well as the absence of accepted thresholds of mutated ctDNA fractions able to definitively predict the responsiveness to EGFR antagonists.

## Author Contributions

VI and FM wrote the manuscript. GP made figures. MT gave a substantial cultural contribute to the work. FS and CP finally revised the text. All authors contributed to the article and approved the submitted version.

## Conflict of Interest

The authors declare that the research was conducted in the absence of any commercial or financial relationships that could be construed as a potential conflict of interest.
